# Recent evolutionary history of *Chrysoperla externa* (Hagen 1861) (Neuroptera: Chrysopidae) in Brazil

**DOI:** 10.1371/journal.pone.0177414

**Published:** 2017-05-16

**Authors:** Adriana C. Morales-Corrêa e Castro, Nara Cristina Chiarini Pena Barbosa

**Affiliations:** 1 Programa de Pós-Graduação em Biociências, Departamento de Biologia, Instituto de Biociências, Letras e Ciências Exatas, Univ. Estadual Paulista “Júlio de Mesquita Filho”, São José do Rio Preto, SP, Brazil; 2 Departamento de Biologia Aplicada à Agropecuária, Faculdade de Ciências Agrárias e Veterinárias, Univ. Estadual Paulista “Júlio de Mesquita Filho”, Jaboticabal, SP, Brazil; National Cheng Kung University, TAIWAN

## Abstract

This work aimed to elucidate the distribution of *Chrysoperla externa* haplotypes and investigate whether it exhibits structure based on genetic composition as opposed to geographic location. The genetic diversity of *C*. *externa*, analyzed by AMOVA using the *COI* and *16S* rRNA genes as mitochondrial markers, showed significant haplotype structure arising from genetic differences that was not associated with sampling location. This was reflected in the network grouping. Bayesian inference showed that haplotype distribution may have its origins in *C*. *externa* divergence into two distinct clades, which dispersed to various locations, and their subsequent diversification. The evolutionary history of *C*. *externa* may include multiple ancestral haplotypes differentiating within the same geographic area to generate the current broad genetic diversity, so that the earlier geographical history has been erased, and now we have highlighted its more recent genetic history.

## Introduction

Green lacewings are insects of the Chrysopidae, a family of 1,413 species and subspecies distributed among 82 genera [1]. *Chrysoperla* Steinmann 1964 comprises 36 lacewing species, four of which are found in Brazil: *Chrysoperla defreitasi* Brooks 1994, *Chrysoperla raimundoi* Freitas & Penny 2001, *Chrysoperla genanigra* Freitas 2003, and *Chrysoperla externa* (Hagen 1861). The last named is one of the most common lacewing species in the Americas and can be found from the southern USA to Argentina [2].

Studies of the biology of *C*. *externa* and its use as a biological control agent have been published since the 1970s [3]. The genetic variation and degree of population structure of *C*. *externa* began to be studied only in 2007, culminating in publication of a study conducted in different agrosystems and seasons using mitochondrial DNA as a molecular marker in specimens from Jaboticabal, São Paulo (SP), Brazil [4]. The specimens of *C*. *externa* showed high haplotype diversity (0.956) with 24 haplotypes in 36 specimens analyzed, and no population structure. The study of population structure of this species in São Paulo State was the main, but not sole, focus of later research. The genetic variability and genetic relationships among populations were estimated by genetic markers *ISSR* and the *COI* gene in 90 specimens from 12 populations in SP. High levels of diversity were observed for both markers, with the highest values found in municipalities having the largest areas of native vegetation [5]. A further 12 populations and 114 specimens were studied in SP, revealing high genetic diversity and lack of geographic structure, suggesting that *C*. *externa* is a single population, regardless of sampling location [6]. The most comprehensive study of *C*. *externa* distribution compared the variability and degree of structure in populations from areas of native vegetation with those from agrosystems, analyzing 122 specimens and 12 previously unstudied locations, primarily Brazilian states. The results showed population expansion in agro-ecosystems, whereas populations in the native environment remained stable over time. Analysis showed significant genetic structure among the sampled groups, which, combined with its within-group absence, corroborated group identities [7].

Although the studies carried out to date have analyzed a sufficient number of specimens for population studies of *C*. *externa*, different groups were sampled, making it difficult to compare parameters and gain a comprehensive understanding of the species evolution. Research using the mitochondrial marker *16S rRNA* gene, as proposed in this study, provides information on population history on a wider time scale than do the *COI* gene and *ISSR* markers previously used [4–7].

The goal of this study was to reveal the genetic structure of *C*. *externa*, combining, for the first time, data on all specimens analyzed for the *COI* gene in different locations and data of specimens from locations not previously analyzed, along with analysis of the *16S rRNA* gene, to investigate whether populations of *C*. *externa* exhibits differentiation based on genetic composition or on the geographic source of the specimens.

## Materials and methods

### Biological materials

This study gathers new specimens of *C*. *externa* and analyzed in previous papers by our group [4, 5 and 7]. We sampled 123 localities in Brazil in the states of São Paulo (110), Minas Gerais (7), Paraná (3), Goiás (1), Rio Grande do Norte (1) and Distrito Federal (1) and at a location in Paraguay; all the localities are georeferenced (S1 Table). Collections were performed from 2005 to 2010 with permission issued by the agency responsible for environmental studies in Brazil—ICMBio-IBAMA (Chico Mendes Institute for Biodiversity Conservation of the Brazilian Institute of the Environment). All the specimens were collected in open areas and on agrosystems borders. No collecting activities occurred in protected areas, natural reserves or state/federal parks, nor did it involve endangered or protected species, as shown in the table of localities (S1 Table). The specimens were stored in absolute ethanol and identified based on external morphological characteristics according to Brooks and Barnard [8] by Dr. Sérgio de Freitas. Currently, the vouchers specimens of this work, together with the other individual specimens collected in the expeditions are deposited in the Coleção de Referência Entomológica (Collection of Entomological Reference) do Departamento de Fitossanidade of the Faculdade de Ciências Agrárias e Veterinárias (FCAV-UNESP), as part of the Neuroptera Collection and open for scientific consultation.

### DNA extraction

DNA was extracted from the thorax, while the head, wings, and abdomen were stored in absolute ethanol also in the Coleção de Referência Entomológica at the Departamento de Fitossanidade of the FCAV-UNESP (S1 Table). The DNA was extracted using the Wizard^®^ Genomic DNA Purification Kit (Promega), following the manufacturer’s protocol and it is stored at the Laboratório de Biologia Evolutiva (LaBE) do Departamento de Biologia Aplicada à Agropecuária—FCAV-UNESP.

### Amplification and sequencing

The mitochondrial genes *COI* and *16S rRNA* were amplified by PCR in a Mastercycler thermal cycler (Eppendorf). The reaction was conducted in a final volume of 25 μL consisting of 12.5 μL of GoTaq^®^ Colorless Master Mix (Promega), 0.4 μM of each of the primers, and 2.5 μL total DNA (~40 ng). The *COI* gene was amplified with primers C1-J-2183 (5’CAACATTTATTTTGATTTTTTGG3’) and TL2-N-3014 (5’TCCATTGCACTAATCTGCCATATTA3’) [9] under the following conditions: initial denaturation at 94°C for 2 minutes followed by 35 cycles of 40 seconds at 94°C, 50 seconds at 55°C, and 1 minute at 72°C; with a final extension phase of 72°C for 10 minutes. The *16S* gene was amplified with primers LR-J-12887 (5’ CCGGTTTGAACTCAGATCATGT 3’) and SR-N-13398b (5’ CRCYTGTTTAWCAAAAACAT 3’) [9] under conditions as follows: initial denaturation at 95°C for 5 minutes followed by 33 cycles of 20 seconds at 93°C, 40 seconds at 50°C, and 2 minutes at 72°C; with a final extension phase of 72°C for 10 minutes. The PCR products were subjected to electrophoresis in 1% agarose gel and stained with ethidium bromide (1ng/mL) to confirm amplification. Each PCR product was purified using the Wizard^®^ SV Gel and PCR Clean-Up System (Promega) according to the manufacturer’s protocol. The products were sequenced using the same primers and amplification conditions. The sequencing reactions were conducted in an ABI 3730 XL DNA Analyzer (Applied Biosystems, Foster City, California, CA) automatic sequencer using the BigDye^®^ Terminator v.3.1 Cycle Sequencing Kit (Perkin-Elmer Applied Biosystems), and the sequences were deposited in the Laboratório de Biologia Evolutiva (LaBE) database at the Departamento de Biologia Aplicada à Agropecuária, FCAV. The haplotypes were deposited in the GenBank database under the accession numbers KJ586656 to KJ586673 and KX099401 to KX099607 (S2 and S3 Tables).

### Data analysis

The sequences were read in Chromas Lite v.2.1.1 [10] and aligned with Muscle Tool Web Services [11]. Descriptive analyses were performed using DnaSP v.5.10.01 [12], and the number of polymorphic sites (S), number of haplotypes (h), haplotype diversity (Hd), nucleotide diversity (π), and average number of nucleotide differences (k) were obtained. Nucleotide composition was calculated by MEGA v.6.06 [13]. The Analysis of Molecular Variance (AMOVA) was applied using Arlequin v.3.5.1.3 [14]. A haplotype network was built using TCS v.1.21 [15]. The presence of population structure was tested for the genes separately using a Bayesian approach with Structure v.2.3.4 [16–19]. Several runs were realized without replicates to estimate the probable K value (clusters) based on the ln(Pr(X|K) value. Twenty-five independent runs were carried out for each K value, ranging from 1 to 8 for the *COI* gene and 1 to 5 for the *16S* gene. For each run, 10,000 iterations were carried out after a burn-in of 10,000 iterations. The assumed parameters for both genes were "no model admixture" because they were haploid mitochondrial genes, and "all frequencies correlated" since the sequences had high similarity [17]. To detect the number of genetically homogeneous groups (K) that best fit the data, we used Evanno implemented [20] in the Structure Harvester website [21]. A graphic representation of the results obtained by the Structure program was created in CLUMPAK beta version [22]. Tajima’s D [23] and Fu’s Fs [24] neutrality tests were performed with concatenated data by DnaSP v.5.10.01 [12], to determine if populations followed a neutral model of evolution with constant population size over time. The saturation of the data was assessed with the DAMBE v.6.0.48 [25] using the Test of substitution saturation by Xia et al. [26, 27]. The phylogenetic reconstruction was performed by Bayesian inference using the package BEAUti and BEAST v.1.8.2 [28] with concatenated data. The substitution model most appropriate for the *COI* gene was GTR+I+G and for *16S* gene was HKY+I, obtained using JModelTest v.2.1.3, utilizing the Bayesian Information Criterion [29]. We performed 150 million MCMC simulations and sampled every 1500 steps, under the lognormal relaxed clock model and a constant-size coalescent tree prior. The species of Chrysopidae, *Chrysoperla defreitase* (*COI*: KX099404; *16S*: KX099549), *Chrysopodes divisa* (*COI*: KX099403; *16S*: KX099548), *Ceraeochrysa cubana* (*COI*: KX099402; *16S*: KX099547), *Leucochrysa cruentata* (*COI*: KX099401; *16S*: KX099546), and Hemerobiidae, *Neuronema laminatum* (NC_028153.1) were used as outgroups. The priors were checked in Tracer v.1.6 [30] and the maximum clade credibility tree was generated using Tree Annotator v.1.8.2 [31], considering the burn-in value to equal 1% of the total trees generated in BEAST. This was viewed and edited in FigTree v.1.4.2 [32].

## Results

### Descriptive statistics

We obtained 477 sequences of 730 bp each for the *COI* gene and 158 haplotypes (S2 Table), and 466 sequences of 488 bp each for the *16S* gene and 58 haplotypes (S3 Table). The concatenated data generated 387 sequences of 1,218 bp each, which presented an average nucleotide composition of 40.3% thymine (T), 14.5% cytosine (C), 32.8% adenine (A), and 12.4% guanine (G). One hundred fifty-eight polymorphic sites (S) were obtained, resulting in 193 haplotypes (h) (S4 Table) and an average haplotype diversity (Hd) of 0.9627. The average number of nucleotide differences (k) and average nucleotide diversity (π) were 3.321 and 0.00274, respectively.

### Population structure

The genetic structure was used to investigate the species *C*. *externa* in a geographic and genetic context. Initially, AMOVA was used to test the structure by locality, considering all populations as a single group. For this grouping, the p-value was not significant for either gene (*COI*: p = 0.69404±0.01436; *16S*: p = 0.56794±0.01391), showing that there was not sufficient evidence to prove that there is genetic structure among geographic populations.

**Fig 1:**
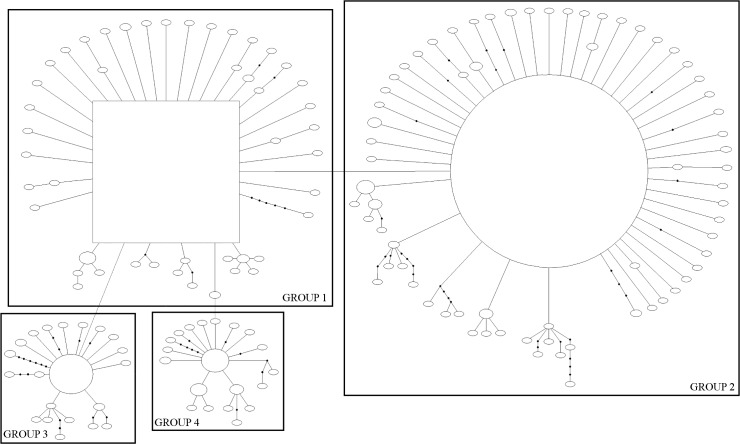
Haplotype network for the *COI* gene of *Chrysoperla externa*. Square represent the ancestral haplotypes. The size of circle reflects the frequency of the haplotypes in the sample. Solid lines correspond to a mutational change connecting two haplotypes with a probability >95%. Small black dots denote missing intermediate haplotypes.

**Fig 2:**
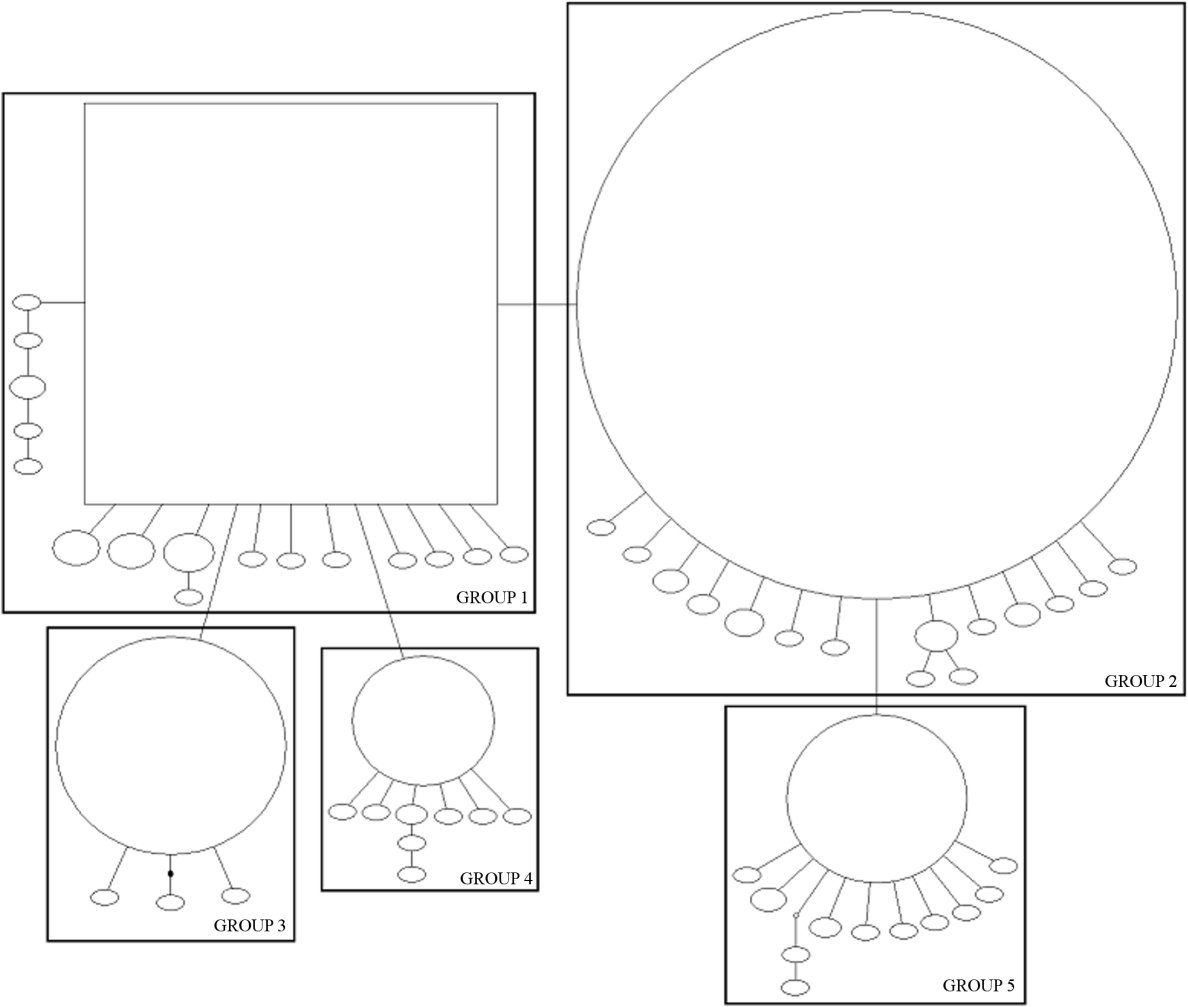
Haplotype network for the *16S* gene of *Chrysoperla externa*. Square represents the ancestral haplotype. The size of circle reflects the frequency of the haplotypes in the sample. Solid lines correspond to a mutational change connecting two haplotypes with a probability >95%. Small black circles denote missing intermediate haplotypes.

The haplotype network for both genes showed well-defined groups. The *COI* gene comprised four groups (Fig 1), and the 16S gene exhibited five groups (Fig 2). All groups contained specimens from different localities; hence grouping based on geographic distribution was not observed (S1–S3 Tables). The distribution of haplotypes was coincident in Group 4, in which the specimens were isolated from the other groups with respect to both genes (S1 Fig and S2 Fig). All specimens in Group 3 of the *16S* gene were also in Group 1 of the *COI* gene. The specimens were not coincident in other groups; specimens belonging to a group in the *COI* gene appeared in different groups in the *16S* gene, and vice-versa (S1 Fig and S2 Fig).

Posteriorly, AMOVA was used to test the structure based on groups defined by the parsimony analysis in TCS. For this grouping, the p-value was significant for both genes (p = 0.00000 ± 0.00000 for both), and the F_ST_ presented high values (*COI*: F_ST_ = 0.55559; *16S*: F_ST_ = 0.75517). The population pairwise F_ST_s also presented high values, demonstrating that Group 4 did not skew this structure (Table 1).

**Table 1:** Pairwise F_ST_s for grouping based on haplotype network constructed by using the statistical parsimony method of *Chrysoperla externa*.

	*COI*				*16S*				
	1	2	3	4	1	2	3	4	5
1	0.00000				0.00000				
2	0.42111	0.00000			0.65494	0.00000			
3	0.43204	0.56469	0.00000		0.65333	0.87631	0.00000		
4	0.69990	0.65796	0.72509	0.00000	0.60208	0.84387	0.86208	0.00000	
5	-	-	-	-	0.74191	0.71340	0.89001	0.83259	0.00000

**Fig 3:**
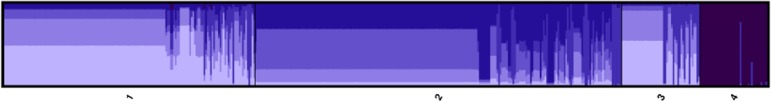
Population structure for grouping based on haplotype network constructed by using the statistical parsimony method for *COI* gene of *Chrysoperla externa*. Each individual is represented by a thin vertical line, which is partitioned into coloured segments that represent the individual’s probability of belonging to the cluster with that colour. Colors represent clusters (K), interpreted as mutational steps from an ancestral haplotype not present in the analysis.

**Fig 4:**
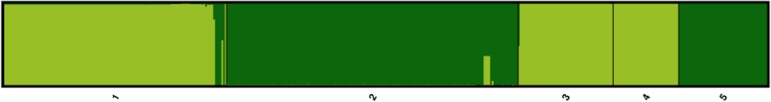
Population structure for grouping based on haplotype network constructed by using the statistical parsimony method for *16S* gene of *Chrysoperla externa*. Each individual is represented by a thin vertical line, which is partitioned into coloured segments that represent the individual’s probability of belonging to the cluster with that colour. Colors represent clusters (K), interpreted as mutational steps from an ancestral haplotype not present in the analysis.

Structure analysis was used to visualize the genetic similarity among individuals and test the presence of population structure. For the *COI* gene, individuals were grouped into six clusters (Figs 1 and 3), which were interpreted as mutational steps occurring from an ancestral haplotype not present in the analysis. Individuals of the Groups 1 and 3 presented similar proportions of the coloured segments that represent the individual’s probability of belonging to the cluster with that colour. Group 2 presented the same clusters as Groups 1 and 3, but in different probability. Group 4 appeared separately, similar to results of TCS, indicating genetic composition distinct from other groups. The *16S* gene was separated into two clusters, with Groups 1, 3, and 4 in a cluster and Groups 2 and 5 in another (Figs 2 and 4).

### Bayesian analysis and neutrality test

**Fig 5:**
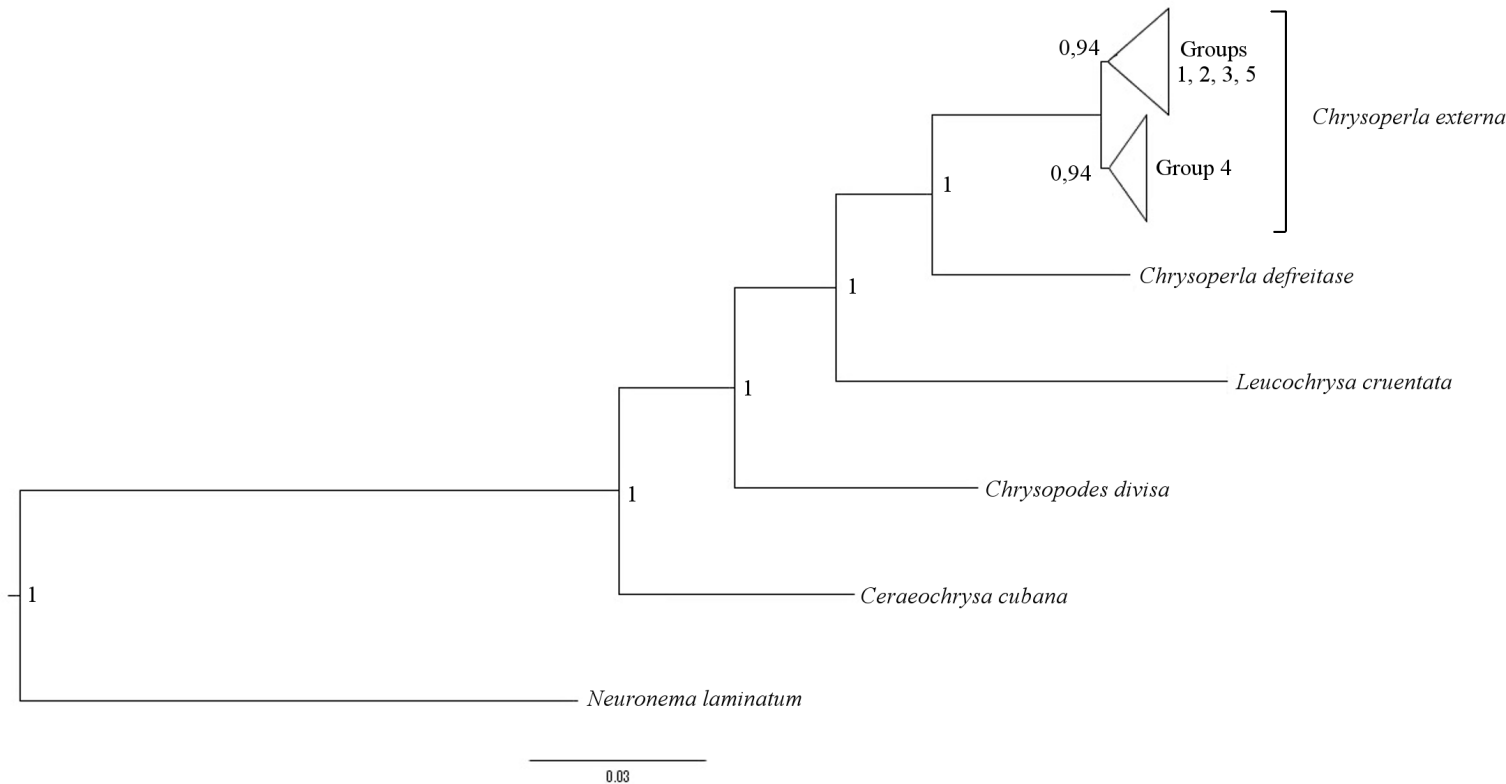
Bayesian inference analysis for the concatenated genes of *Chrysoperla externa*. The node type represents Bayesian posterior probability.

Bayesian inference analysis was performed to obtain the probable relationship between haplotypes of *C*. *externa*. The saturation test showed little saturation, indicating that the date was appropriate for phylogenetic analysis (Iss<ISSc; p < 0.05). *Chrysoperla externa* split into two distinct clades with 100% posterior probability, one comprised of individuals appearing only in the TCS Group 4 and a second clade including individuals of other groups (Fig 5). The clade comprising Groups 1, 2, 3, and 5 showed internal clades that were not monophyletic, while the Group 4 clade was monophyletic and showed more recent divergence.

Based on the Bayesian inference separation, the neutrality test was conducted for Group 4 separately from the remaining groups. The observed values of Tajima’s D and Fu’s F_S_ neutrality tests were -2.27254 (p < 0.01) and -17.508 (p = 0.000), respectively, for Group 4, and -2.68706 (p < 0.01) and -315.397 (p = 0.000), respectively, for other groups.

## Discussion

Our results demonstrated that *C*. *externa* has significant haplotype structure arising from genetic differences not shown to be influenced by present geography. Almost all the 158 *COI* haplotypes and 58 *16S* haplotypes were found in all sampled locations.

The AMOVA demonstrated that haplotype differences among locations were not significant, showing that the distances between sampling sites, in Brazil and Paraguay, did not act as a barrier, and produced no geographic differentiation. This would be a necessary premise for the phylogeographic inference test, which could assess the haplotype and geographic association [33]. This did not occur in the present or in previous studies of *C*. *externa* [4–7]. However, the presence of distinct groups was notable in the haplotype networks (Figs 1 and 2) as a consequence of the level of difference/mutation among them (S1 Supporting Information).

Phylogeographic studies use the clades formed in the haplotype network to test the hypotheses of genetic/geographical association and several studies show a correspondence between the geographic distribution and network topology [34–43]. Therefore, we used the groups formed in the network to assess by AMOVA the relationship between the distinct haplotypic groups, and the analysis revealed significant differences in both analyzed genes. Hence, the haplotypes showed genetic differences sufficient to be allocated into groups, and that different haplotypic groups coexisted in a location.

Bayesian inference performed on the concatenated data suggested that there was initially a divergence of *C*. *externa* haplotypes into two distinct clades, which diversified posteriorly (Fig 5). This clear separation of haplotypes into clades of *C*. *externa* does not correspond to a geographic structure, as confirmed by AMOVA. The clade with the most recent diversification was composed exclusively of individuals from Group 4 in both gene haplotype networks. In both analyzed genes, these 34 individuals showed greater similarity to one another than to the other groups.

In the structure analysis of the *COI* gene using Structure (Fig 3), the union of Groups 1, 2, and 3 indicated by the presence of at least one cluster in common, and the segregation of Group 4, reinforced the results of the Bayesian analysis (Fig 5) for the divergence into two clades. For *16S*, the structure analysis showed the separation into only two clusters (Fig 4) with Group 4 remaining associated with Groups 1 and 3. This can be attributed to the fact that the *16S* gene is conserved and non-coding, so that the primary difference observed between the two clusters is the insertion of one or two bases at positions 173 and 174 (S1 Supporting Information). Thus, the phylogenetic information that shows the initial divergence may have been diluted in the structure analysis, since it does not include the ancestral state present in the outgroup [44, 45].

The population expansion followed by diversification of haplotypes can be inferred by the star-like network topology of both genes, as well as by the neutrality tests. The networks displayed two more frequent haplotypes that were considered ancestral (S2 Table and S3 Table) from which many haplotypes were derived, suggesting that most of these haplotypes emerged later [46]. The results of Tajima's D and Fu's F_S_ neutrality tests were negative, indicating an excess of rare alleles within the population, which may suggest population expansion [47, 48].

These results suggest that the evolutionary history of *C*. *externa* may include the existence of multiple ancestral haplotypes within a same geographic area. However, it is possible to infer that this pattern found for *C*. *externa* can be derived from a strong anthropic action, involving exchanges of plant seedlings with the petiolate eggs between agricultural producers. Thus, this agricultural practice may have promoted homogenization in the populations trough the introduction of haplotypes from other regions with distinct genetic characteristics, covering up a possible regional differentiation. As a result of this introduction, it is currently observed a pattern of co-existence of multiple ancestral haplotypes in the same place, but belonging to groups excessively genetically distinct to be sharing the same area of occurrence in a biogeographic point of view, and thus indicating that these haplotypes have had an allopatric differentiation.

Therefore, it is possible that the earlier geographical history of *C*. *externa* has been erased, and now we have highlighted its more recent genetic history.

## Supporting information

S1 Fig Haplotype network for the *COI* gene of *Chrysoperla externa*. Square represents the ancestral haplotype. The size of circles reflects the frequency of the haplotypes in the sample. Haplotype names correspond to names in S1 Table. Solid lines correspond to a mutational change connecting two haplotypes with probability >95%. Small circles denote missing intermediate haplotypes.
(TIF)

S2 Fig Haplotype network for the *16S* gene of *Chrysoperla externa*. Square represents the ancestral haplotype. The size of circles reflects the frequency of the haplotypes in the sample. Haplotype names correspond to names in S1 Table. Solid lines correspond to a mutational change connecting two haplotypes with probability >95%. Small circles denote missing intermediate haplotypes.
(TIF)

S1 Supporting Information Variable sites of haplotypes in concatenated genes of *Chrysoperla externa*. Positions from 1 to 730 are for the *COI* gene, and from 731 to 1218 are for the *16S* gene.
(TXT)

S1 Table Sampled localities. Sampling localities, geographic coordinates, number of specimens analyzed (*COI*/*16S*), and *Chrysoperla externa* voucher number.
(PDF)

S2 Table Haplotype list of the *COI* gene. Identification code (ID) of haplotypes for *COI* gene; number of specimens containing each haplotype; GenBank accession number; *Chrysoperla externa* voucher number.
(PDF)

S3 Table Haplotype list of the *16S* gene. Identification code (ID) of haplotypes for *16S* gene; number of specimens containing each haplotype; GenBank accession number; *Chrysoperla externa* voucher number.
(PDF)

S4 Table Haplotype list of concatenated genes. Identification code (ID) of haplotypes for concatenated data (*COI* and *16S* genes); number of specimens containing each haplotype; *Chrysoperla externa* voucher number.
(PDF)
