# Supplementary figures and images for: Recent evolutionary history of Chrysoperla externa (Hagen 1861) (Neuroptera: Chrysopidae) in Brazil
Source: PLoS One. 2017 May 16;12(5):e0177414. doi: 10.1371/journal.pone.0177414 (PMC5433706; doi:10.1371/journal.pone.0177414)

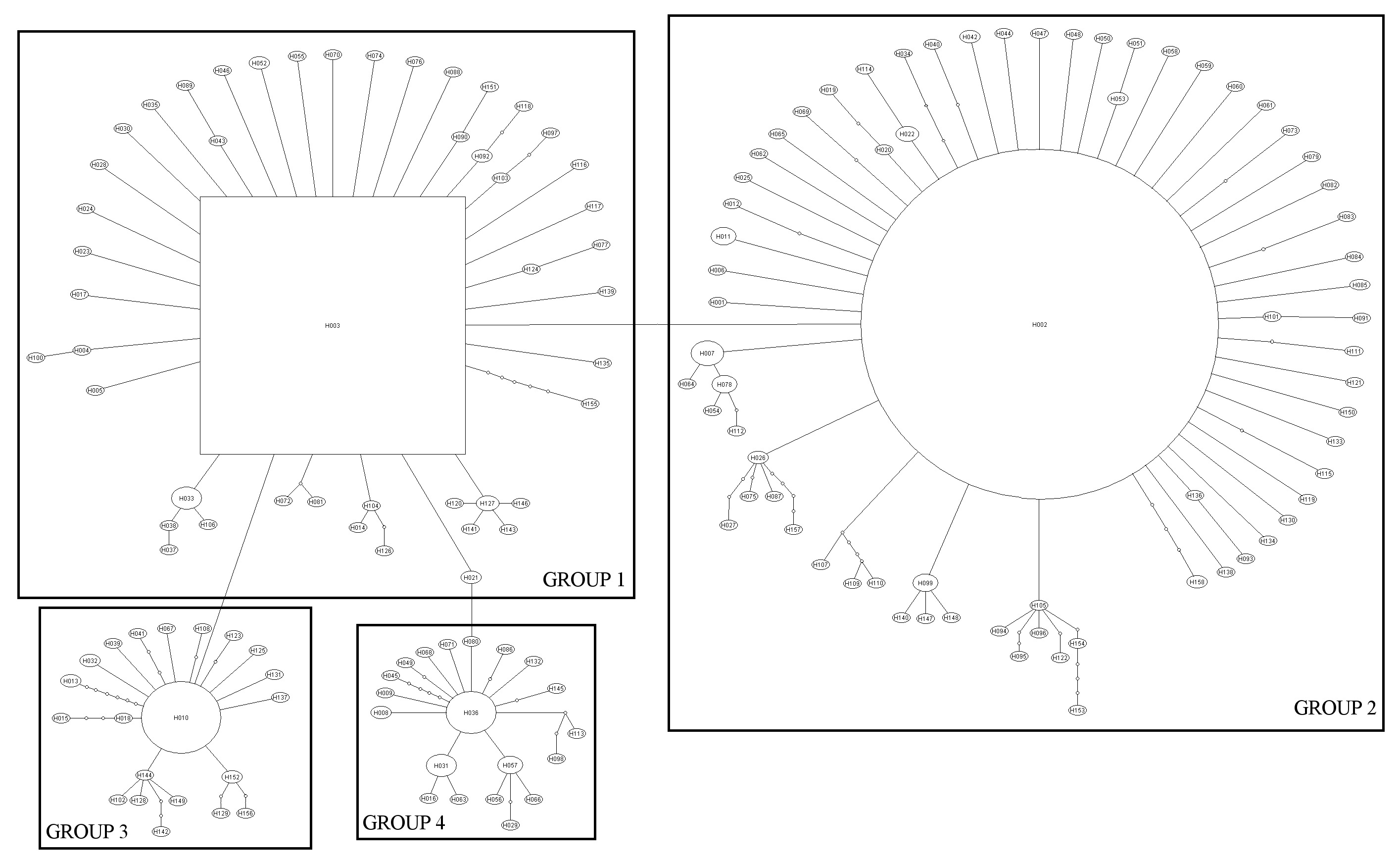

Supplement: S1 Fig — Square represents the ancestral haplotype. The size of circles reflects the frequency of the haplotypes in the sample. Haplotype names correspond to names in S1 Table. Solid lines correspond to a mutational change connecting two haplotypes with probability >95%. Small circles denote missing intermediate haplotypes. (TIF) [file pone.0177414.s001.tif]
