# Supplementary material for: Recent evolutionary history of Chrysoperla externa (Hagen 1861) (Neuroptera: Chrysopidae) in Brazil
Source: PLoS One. 2017 May 16;12(5):e0177414. doi: 10.1371/journal.pone.0177414 (PMC5433706; doi:10.1371/journal.pone.0177414)
Supplement: S1 Table — Sampling localities, geographic coordinates, number of specimens analyzed (COI/16S), and Chrysoperla externa voucher number. (PDF) [file pone.0177414.s004.pdf]

S1 Table. Sampled localities. Sampling localities, geographic coordinates, number of specimens analyzed (*COI*/*16S*), and *Chrysoperla externa* voucher number.

Localities	State	Geographic coordinates	n (COI/16S)	Voucher
Águas da Prata	SP	-21:56:13 -46:43:01	9/3	143-146; 885- 890
Agudos	SP	-22:28:08 -48:59:16	1/4	127-130
Alambari	SP	-23:33:03 -47:53:56	3/3	420-422
Altair	SP	-20:31:26 -49:03:32	3/4	423-426
Alvinlândia	SP	-22:26:38 -49:45:46	1/1	427
Analândia	SP	-22:07:33 -47:39:46	3/4	428-431
Andradas	MG	-22:04:04 -46:34:08	1/1	826
Andradina	SP	-20:53:45 -51:22:44	9/3	75-78; 875-884
Araras	SP	-22:21:25 -47:23:02	2/4	432-435
Arealva	SP	-22:01:44 -48:54:39	4/4	436-439
Avaí	SP	-22:08:49 -49:19:58	3/3	83-86
Avaré	SP	-23:05:56 -48:55:33	2/5	440-444
Balbinos	SP	-21:54:00 -49:21:25	2/2	445-446
Bálsamo	SP	-20:44:06 -49:35:02	0/3	447-449
Bariri	SP	-22:04:26 -48:44:24	2/4	450-453
Barretos	SP	-20:33:25 -48:34:04	4/3	123-125; 892-896
Bernardino de Campos	SP	-23:00:46 -49:28:26	1/4	454-457
Boa Esperança do Sul	SP	-21:59:34 -48:23:27	3/4	458-461
Borborema	SP	-21:37:12 -49:04:26	2/4	462-465
Borebi	SP	-22:34:08 -48:58:15	2/4	466-469
Botucatu	SP	-22:53:09 -48:26:42	2/4	470-473
Brasília	DF	-15:46:48 -47:55:48	0/1	802
Brotas	SP	-22:17:02 -48:07:37	7/0	898-905
Cabrália Paulista	SP	-22:27:21 -49:20:16	3/4	474-477
Cafelândia	SP	-21:48:10 -49:36:36	3/3	79-82
Cajobi	SP	-20:52:48 -48:48:32	1/1	478
Cândido Rodrigues	SP	-21:19:30 -48:37:51	2/2	479-480
Casa Branca	SP	-21:46:26 -47:05:09	3/4	481-484
Catiguá	SP	-21:02:56 -49:03:28	4/4	485-488
Conchal	SP	-22:19:48 -47:10:22	6/3	91-93; 908-913
Cordeirópolis	SP	-22:28:55 -47:27:25	3/3	490-492
Corumbataí	SP	-22:13:12 -47:37:33	4/3	493-496
Delfim Moreira	MG	-22:30:32 -45:16:48	5/6	1160-1165
Descalvado	SP	-21:54:14 -47:37:08	4/4	497-500
Dobrada	SP	-21:31:01 -48:23:38	2/3	502-504
Duartina	SP	-22:24:50 -49:24:14	3/4	506-508
Embaúba	SP	-20:58:58 -48:50:09	4/4	509-512
Engenheiro Coelho	SP	-22:29:16 -47:12:54	3/3	514-516
Fernando Prestes	SP	-21:15:50 -48:41:06	3/4	517-520
Frutal	MG	-20:01:30 -48:56:27	4/4	521-524
Gastão Vidigal	SP	-20:47:56 -50:11:13	3/4	107-110

Localities	State	Geographic coordinates	n (COI/16S)	Voucher
Getulina	SP	-21:47:56 -49:55:44	4/3	525-528
Guaimbê	SP	-21:54:36 -49:53:49	4/4	529-532
Guapiaçu	SP	-20:47:42 -49:13:12	4/4	534-537
Guarantã	SP	-21:53:42 -49:35:24	3/4	538-541
Iacanga	SP	-21:53:24 -49:01:30	4/4	542-545
Ibirá	SP	-21:04:48 -49:14:27	1/1	546
Ibitinga	SP	-21:45:28 -48:49:44	1/1	547
Icém	SP	-20:20:31 -49:11:42	2/3	548-550
Ipameri	GO	-17:43:19 -48:09:36	11/12	533; 737-767
Itaju	SP	-21:58:51 -48:48:18	1/1	551
Itápolis	SP	-21:35:45 -48:48:46	3/3	147-150
Jaboticabal	SP	-21:15:18 -48:19:19	42/15	1-70
Jaci	SP	-20:52:55 -49:34:12	1/1	552
Jales	SP	-20:16:08 -50:32:45	4/2	553-554; 953-958
José Bonifácio	SP	-21:03:10 -49:41:16	2/2	555-556
Júlio Mesquita	SP	-22:00:32 -49:47:13	3/4	557-560
Leme	SP	-22:11:09 -47:23:24	2/4	561-564
Lins	SP	-21:40:44 -49:44:34	3/3	565-567
Lucianópolis	SP	-22:25:51 -49:31:22	4/4	568-571
Luís Antônio	SP	-21:33:18 -47:42:14	7/4	103-106; 921-926
Lupércio	SP	-22:24:54 -49:49:01	1/1	572
Marília	SP	-22:12:50 -49:56:45	2/4	573-576
Maringá	PR	-23:25:30 -51:56:20	10/12	857-868
Matão	SP	-21:36:10 -48:21:57	4/4	577-580
Meridiano	SP	-20:21:32 -50:10:22	1/1	581
Mirassolândia	SP	-20:37:01 -49:27:50	1/1	582
Mococa	SP	-21:28:04 -47:00:18	3/3	583-586
Mogi Mirim	SP	-22:25:55 -46:57:28	3/3	587-590
Monte Aprazível	SP	-20:46:22 -49:42:50	7/4	99-102; 928-930
Mossoró	RN	-5:11:16 -37:20:38	4/4	827-830
Nova Aliança	SP	-21:00:57 -49:29:45	1/1	591
Nova Europa	SP	-21:46:40 -48:33:39	4/4	592-595
Nova Granada	SP	-20:32:02 -49:18:50	3/3	596-599
Novais	SP	20:59:31 -48:55:08	4/4	600-603
Ocauçu	SP	-22:26:20 -49:55:22	1/1	604
Olímpia	SP	-20:44:13 -48:54:54	4/4	605-608
Onda Verde	SP	-20:36:50 -49:17:56	4/4	609-612
Palestina	SP	-20:23:24 -49:25:58	0/3	131-134
Parisi	SP	-20:18:14 -50:00:54	0/3	119-121
Paulistânia	SP	-22:34:40 -49:24:10	3/4	613-616
Pedranópolis	SP	-20:14:52 -50:06:36	4/4	617-620
Pedro Juan Caballero	Paraguai	-22:31:46 -55:44:51	16/16	997-1012
Pereira Barreto	SP	-20:38:16 -51:06:32	2/4	139-142

Localities	State	Geographic coordinates	n (COI/16S)	Voucher
Pirajuí	SP	-21:59:56 -49:27:25	4/4	621-624
Pirassununga	SP	-21:59:45 -47:25:33	4/4	625-628
Piratininga	SP	-22:24:46 -49:08:06	2/4	629-632
Planura	MG	-20:08:16 -48:42:07	4/4	633-636
Poloni	SP	-20:47:06 -49:49:26	2/2	641-642
Pongaí	SP	-21:44:09 -49:22:01	4/3	637-640
Potirendaba	SP	-21:02:34 -49:22:37	1/2	643-644
Prata	MG	-19:18:25 -48:55:26	2/3	645-647
Presidente Bernardes	SP	-22:00:21 -51:33:10	2/2	649-651; 951
Presidente Prudente	SP	-22:07:33 -51:23:20	20/17	71-73; 653-655; 781-797; 934-944
Reginópolis	SP	-21:53:16 -49:13:30	2/3	656-658
Rifaina	SP	-20:04:51 -47:25:15	4/0	978-988
Rincão	SP	-21:35:13 -48:04:15	2/4	659-662
Rio Claro	SP	-22:24:39 -47:33:39	4/4	663-666
Riolândia	SP	-19:59:24 -49:40:51	1/1	667
Sales	SP	-21:20:27 -49:29:06	1/1	672
Santa Albertina	SP	-20:01:55 -50:43:40	4/4	673-676
Santa Cruz do Rio Pardo	SP	-22:53:56 -49:37:58	2/2	682-684
Santa Ernestina	SP	-21:27:46 -48:23:27	3/3	686-688
Santa Salete	SP	-20:14:42 -50:41:16	3/4	677-680
Santana da Ponte Pensa	SP	-20:15:10 -50:47:49	4/4	95-98
Santo Antônio do Aracanguá	SP	-20:56:13 -50:29:45	3/4	689-692
São Carlos	SP	-22:01:04 -47:53:27	1/1	111
São Mateus do Sul	PR	-25:52:26 -50:22:58	10/10	809-819
São Sebastião do Paraíso	MG	-20:55:01 -46:59:27	13/12	803-808; 850-856
Sarandi	PR	-23:26:38 -51:52:26	11/11	820-825; 840-844
Sarutaiá	SP	-23:16:22 -49:28:48	10/4	87-90, 964-975
Severínia	SP	-20:48:32 -48:48:10	2/3	135-137
Tabapuã	SP	-20:57:50 -49:01:55	2/3	694-696
Tabatinga	SP	-21:43:01 -48:41:16	1/1	697
Tambaú	SP	-21:42:18 -47:16:26	4/3	698-701
Tanabi	SP	-20:37:33 -49:38:56	3/3	702-704
Taquaritinga	SP	-21:24:21 -48:30:18	4/3	705-708
Três Pontas	MG	-21:22:01 -45:30:46	10/10	1130-1139
Turmalina	SP	-20:03:07 -50:28:33	2/2	709-710
Ubarana	SP	-21:09:57 -49:43:04	1/2	711-712
Ubirajara	SP	-22:31:37 -49:39:46	2/4	115-118
Uchoa	SP	-20:57:10 -49:10:30	1/1	713
Uru	SP	-21:47:02 -49:16:51	4/4	714-717
Zacarias	SP	-21:03:07 -50:03:03	4/4	718-721
