# Supplementary material for: Recent evolutionary history of Chrysoperla externa (Hagen 1861) (Neuroptera: Chrysopidae) in Brazil
Source: PLoS One. 2017 May 16;12(5):e0177414. doi: 10.1371/journal.pone.0177414 (PMC5433706; doi:10.1371/journal.pone.0177414)
Supplement: S2 Table — Identification code (ID) of haplotypes for COI gene; number of specimens containing each haplotype; GenBank accession number; Chrysoperla externa voucher number. (PDF) [file pone.0177414.s005.pdf]

S2 Table. Haplotype list of the *COI* gene. Identification code (ID) of haplotypes for *COI* gene; number of specimens containing each haplotype; GenBank accession number; *Chrysoperla externa* voucher number.

ID	n	access number	voucher
H001	1	KX099405	01
H002	138	KJ586665	02, 03, 07, 10, 12, 17, 22, 30, 32, 41, 48, 50, 54, 75, 78, 79, 82, 83, 90, 93, 95, 98, 100, 102-105, 420, 421, 425, 429, 440, 446, 459, 460, 470, 477, 480, 484, 486, 492, 497, 502, 503, 510, 515, 518, 519, 524, 530, 537, 538, 543, 547, 549, 552, 553, 555, 556, 559, 565, 566, 569, 572, 575, 577, 581, 583, 584, 586, 587, 592, 593, 595, 600, 603, 604, 606, 623, 624, 626, 628, 630, 634-636, 638, 639, 647, 653, 656, 662, 665, 666, 672, 694, 699, 703, 707, 708, 712, 715, 717, 721, 738, 742, 817, 827, 828, 840, 843, 844, 850, 854, 856, 865, 875, 886, 890, 892, 899, 904, 922, 923, 926, 928, 934, 937, 942, 944, 954, 958, 972, 974, 978, 998, 1131, 1133
H003	101	KJ586660	04, 16, 28, 34, 61, 63, 70, 76, 81, 84, 86, 88, 91, 101, 109, 124, 125, 139, 146, 150, 430, 431, 435, 438, 442, 452, 455, 478, 483, 485, 493, 494, 496, 499, 509, 512, 516, 522, 523, 529, 532, 539, 542, 546, 567, 590, 598, 607, 612, 614, 616, 622, 625, 627, 633, 637, 640, 641, 646, 651, 663, 664, 675, 676, 678, 682, 687, 692, 696, 698, 701, 702, 706, 710, 713, 716, 739-741, 765, 785, 808, 822, 826, 829, 841, 876, 884, 896, 898, 905, 913, 929, 935, 951, 953, 970, 985, 987, 988, 1138
H004	1	KX099406	05
H005	1	KX099407	06
H006	1	KX099408	08
H007	7	KX099409	09, 520, 573, 580, 642, 686, 855
H008	2	KX099410	11, 719
H009	1	KX099411	13
H010	26	KJ586666	15, 18, 26, 65, 66, 111, 128, 136, 427, 468, 488, 495, 498, 506, 536, 561, 620, 659, 683, 690, 705, 763, 797, 878, 887, 973
H011	4	KX099412	19, 467, 526, 591
H012	1	KX099413	20
H013	2	KX099414	21, 47
H014	1	KX099415	25
H015	1	KX099416	27
H016	1	KX099417	31
H017	1	KX099418	42
H018	1	KX099419	67
H019	1	KX099420	68
H020	1	KX099421	73
H021	2	KJ586656	77, 697
H022	3	KX099422	89, 621, 674
H023	1	KX099423	92
H024	1	KX099424	96
H025	1	KX099425	97
H026	2	KJ586657	99, 853
H027	1	KX099426	108
H028	1	KX099427	110
H029	1	KX099428	117
H030	1	KX099429	118
H031	6	KX099430	137, 508, 588, 599, 718, 1165
H032	2	KX099431	142, 608
H033	6	KJ586658	143, 451, 667, 704, 709, 787
H034	1	KJ586659	145
H035	1	KX099432	148
H036	14	KJ586664	149, 422, 434, 476, 557, 605, 617, 631, 806, 877, 885, 889, 902, 975
H037	1	KX099433	423
H038	1	KX099434	426
H039	1	KX099435	436
H040	1	KX099436	437
H041	1	KX099437	439
H042	1	KX099438	445, 594
H043	1	KX099439	461
H044	1	KX099440	462
H045	1	KX099441	464
H046	1	KX099442	473

ID	n	access number	voucher
H047	1	KX099443	475
H048	1	KX099444	479
H049	1	KX099445	482
H050	1	KX099446	487
H051	1	KX099447	490
H052	2	KX099448	491, 571
H053	2	KX099449	500, 511
H054	1	KX099450	507
H055	1	KX099451	514
H056	1	KX099452	521
H057	4	KX099453	525, 610, 673, 737
H058	1	KX099454	527
H059	1	KX099455	528
H060	1	KX099456	531
H061	1	KX099457	533
H062	1	KX099458	534
H063	1	KX099459	535
H064	1	KX099460	541
H065	1	KX099461	544
H066	1	KX099462	545
H067	1	KX099463	550
H068	1	KX099464	551
H069	1	KX099465	558
H070	1	KX099466	562
H071	1	KX099467	568
H072	1	KX099468	570
H073	1	KX099469	578
H074	1	KX099470	579
H075	1	KX099471	582
H076	1	KX099472	596
H077	1	KX099473	601
H078	4	KX099474	602, 619, 643, 679
H079	1	KX099475	609
H080	1	KX099476	611
H081	1	KX099477	613
H082	1	KX099478	618
H083	1	KX099479	657
H084	1	KX099480	677
H085	2	KJ586672	688, 921
H086	1	KX099481	691
H087	1	KX099482	700
H088	1	KX099483	714
H089	1	KX099484	720
H090	1	KX099485	764
H091	1	KX099486	767
H092	2	KX099487	781, 782
H093	1	KX099488	786
H094	1	KX099489	788
H095	1	KX099490	791
H096	1	KJ586661	792
H097	1	KJ586662	794
H098	1	KJ586663	795
H099	4	KX099491	796, 866, 1008, 1010
H100	1	KX099492	803
H101	1	KX099493	804
H102	1	KX099494	805
H103	1	KX099495	807
H104	1	KX099496	809
H105	1	KX099497	811
H106	1	KX099498	812
H107	1	KX099499	813
H108	1	KX099500	814
H109	1	KX099501	815
H110	1	KX099502	816

ID	n	access number	voucher
H111	1	KX099503	818
H112	1	KX099504	819
H113	1	KX099505	820
H114	1	KX099506	821
H115	1	KX099507	823
H116	1	KX099508	824
H117	1	KX099509	825
H118	1	KX099510	830
H119	1	KX099511	842
H120	1	KX099512	851
H121	1	KX099513	852
H122	1	KX099514	859
H123	1	KX099515	860
H124	1	KX099516	861
H125	1	KX099517	862
H126	1	KX099518	863
H127	3	KX099519	864, 868, 1003
H128	1	KX099520	867
H129	1	KJ586667	888
H130	1	KJ586669	901
H131	1	KX099521	903
H132	1	KJ586670	908
H133	1	KJ586671	909
H134	1	KJ586673	930
H135	1	KX099522	943
H136	1	KX099523	964
H137	1	KX099524	969
H138	1	KX099525	997
H139	1	KX099526	999
H140	1	KX099527	1000
H141	1	KX099528	1001
H142	1	KX099529	1002
H143	1	KX099530	1004
H144	1	KX099531	1005
H145	1	KX099532	1006
H146	1	KX099533	1007
H147	1	KX099534	1009
H148	1	KX099535	1011
H149	1	KX099536	1012
H150	1	KX099537	1130
H151	1	KX099538	1132
H152	2	KX099539	1134, 1139
H153	1	KX099540	1135
H154	1	KX099541	1136
H155	1	KX099542	1137
H156	1	KX099543	1160
H157	1	KX099544	1161
H158	2	KX099545	1162, 1163
