# Supplementary material for: Recent evolutionary history of Chrysoperla externa (Hagen 1861) (Neuroptera: Chrysopidae) in Brazil
Source: PLoS One. 2017 May 16;12(5):e0177414. doi: 10.1371/journal.pone.0177414 (PMC5433706; doi:10.1371/journal.pone.0177414)
Supplement: S3 Table — Identification code (ID) of haplotypes for 16S gene; number of specimens containing each haplotype; GenBank accession number; Chrysoperla externa voucher number. (PDF) [file pone.0177414.s006.pdf]

S3 Table. Haplotype list of the *16S* gene. Identification code (ID) of haplotypes for *16S* gene; number of specimens containing each haplotype; GenBank accession number; *Chrysoperla externa* voucher number.

ID	n	access number	voucher
H001	151	KX099550	01, 02, 03, 05, 07, 67, 73, 75, 82, 83, 86, 89, 93, 97, 98, 99, 100, 103, 104, 105, 108, 111, 121, 123, 130, 131, 133, 134, 136, 145, 147, 421, 425, 427, 429, 436, 437, 439, 441, 450, 467, 471, 475, 477, 480, 481, 484, 488, 495, 498, 499, 500, 506, 510, 520, 529, 532, 534, 536, 537, 538, 544, 547, 549, 550, 553, 554, 555, 556, 558, 559, 560, 569, 572, 575, 577, 580, 581, 583, 587, 591, 594, 595, 602, 603, 608, 609, 626, 628, 629, 630, 642, 643, 644, 647, 649, 653, 654, 656, 660, 661, 662, 665, 666, 667, 672, 677, 683, 686, 688, 689, 692, 694, 695, 705, 710, 712, 715, 738, 741, 742, 786, 793, 794, 804, 805, 817, 827, 840, 842, 844, 852, 853, 854, 856, 998, 999, 1000, 1002, 1003, 1005, 1008, 1010, 1011, 1012, 1133, 1135, 1139, 1160, 1162, 1164
H002	1	KX099551	04
H003	1	KX099552	06
H004	102	KX099553	08, 09, 66, 77, 78, 87, 96, 101, 102, 109, 115, 119, 128, 129, 139, 142, 420, 432, 433, 443, 446, 453, 462, 468, 470, 472, 486, 487, 490, 503, 507, 511, 518, 527, 528, 531, 533, 539, 548, 552, 561, 564, 566, 571, 573, 582, 584, 586, 590, 592, 600, 601, 615, 618, 619, 620, 621, 623, 625, 634, 635, 636, 639, 651, 658, 664, 679, 680, 684, 690, 698, 700, 717, 721, 763, 767, 784, 787, 791, 792, 796, 797, 813, 816, 818, 819, 821, 823, 828, 843, 850, 858, 860, 862, 864, 865, 866, 867, 868, 997, 1131, 1163
H005	6	KX099554	10, 79, 492, 604, 638, 703
H006	31	KX099555	11, 13, 71, 117, 137, 149, 422, 434, 444, 464, 482, 508, 521, 525, 535, 545, 557, 599, 605, 610, 611, 631, 645, 655, 673, 711, 718, 737, 820, 1006, 1165
H007	6	KX099556	12, 95, 519, 530, 1009, 1136
H008	54	KX099557	81, 84, 107, 118, 120, 124, 125, 135, 150, 430, 442, 452, 461, 465, 466, 469, 473, 478, 483, 491, 494, 509, 516, 517, 522, 542, 546, 562, 622, 632, 641, 646, 663, 696, 697, 702, 713, 714, 716, 765, 766, 782, 802, 810, 822, 824, 825, 826, 829, 851, 863, 1001, 1007, 1138
H009	41	KX099558	88, 91, 127, 143, 423, 424, 426, 431, 438, 445, 449, 451, 458, 485, 496, 504, 512, 515, 523, 576, 578, 598, 606, 613, 614, 633, 675, 676, 678, 701, 704, 709, 740, 781, 812, 830, 841, 857, 859, 1004, 1132
H010	1	KX099559	90
H011	7	KX099560	92, 110, 579, 674, 720, 861, 1161
H012	3	KX099561	106, 565, 574
H013	2	KX099562	116, 455
H014	4	KX099563	140, 479, 707, 815
H015	1	KX099564	141
H016	3	KX099565	146, 616, 739
H017	1	KX099566	428
H018	1	KX099567	435
H019	1	KX099568	440
H020	5	KX099569	447, 497, 563, 788, 1134
H021	1	KX099570	448
H022	1	KX099571	454
H023	1	KX099572	456
H024	3	KX099573	457, 706, 807
H025	1	KX099574	459
H026	1	KX099575	460
H027	1	KX099576	463
H028	3	KX099577	474, 505, 593
H029	2	KX099578	476, 691
H030	2	KX099579	502, 596
H031	1	KX099580	514
H032	1	KX099581	524
H033	1	KX099582	540
H034	1	KX099583	541
H035	1	KX099584	543
H036	1	KX099585	551
H037	1	KX099586	567
H038	1	KX099587	568
H039	1	KX099588	570
H040	1	KX099589	588
H041	1	KX099590	607

ID	n	access number	voucher
H042	1	KX099591	612
H043	1	KX099592	617
H044	1	KX099593	624
H045	1	KX099594	627
H046	1	KX099595	640
H047	1	KX099596	657
H048	1	KX099597	659
H049	1	KX099598	687
H050	1	KX099599	719
H051	1	KX099600	764
H052	1	KX099601	806
H053	1	KX099602	808
H054	1	KX099603	809
H055	1	KX099604	811
H056	1	KX099605	855
H057	1	KX099606	1130
H058	1	KX099607	1137
