# Supplementary material for: Recent evolutionary history of Chrysoperla externa (Hagen 1861) (Neuroptera: Chrysopidae) in Brazil
Source: PLoS One. 2017 May 16;12(5):e0177414. doi: 10.1371/journal.pone.0177414 (PMC5433706; doi:10.1371/journal.pone.0177414)
Supplement: S4 Table — Identification code (ID) of haplotypes for concatenated data (COI and 16S genes); number of specimens containing each haplotype; Chrysoperla externa voucher number. (PDF) [file pone.0177414.s007.pdf]

S4 Table. Haplotype list of concatenated genes. Identification code (ID) of haplotypes for concatenated data (*COI* and *16S* genes); number of specimens containing each haplotype; *Chrysoperla externa* voucher number.

ID	n	voucher
HAP_1	1	01
HAP_2	59	02, 03, 07, 75, 82, 83, 93, 98, 100, 103, 104, 105, 421, 425, 429, 477, 480, 484, 510, 537, 538, 547, 549, 553, 555, 556, 559, 569, 572, 575, 577, 581, 583, 587, 595, 603, 626, 628, 630, 647, 653, 656, 662, 665, 666, 672, 694, 712, 715, 738, 742, 817, 827, 840, 844, 854, 856, 998, 1133
HAP_3	1	04
HAP_4	1	05
HAP_5	1	06
HAP_6	1	08
HAP_7	2	09, 573
HAP_8	6	10, 79, 492, 604, 638, 703
HAP_9	1	11
HAP_10	4	12, 95, 519, 530
HAP_11	1	13
HAP_12	8	66, 128, 468, 561, 620, 690, 763, 797
HAP_13	1	67
HAP_14	1	73
HAP_15	1	77
HAP_16	26	78, 102, 420, 446, 470, 486, 503, 518, 552, 566, 584, 586, 592, 600, 623, 634, 635, 636, 639, 717, 721, 828, 843, 850, 865, 1131
HAP_17	29	81, 84, 124, 125, 150, 430, 442, 452, 478, 483, 494, 509, 516, 522, 542, 546, 622, 641, 646, 663, 696, 702, 713, 716, 765, 822, 826, 829, 1138
HAP_18	7	86, 499, 529, 532, 692, 710, 741
HAP_19	17	88, 91, 431, 438, 485, 496, 512, 523, 598, 614, 633, 675, 676, 678, 701, 740, 841
HAP_20	1	89
HAP_21	1	90
HAP_22	1	92
HAP_23	1	96
HAP_24	1	97
HAP_25	2	99, 853
HAP_26	9	101, 109, 139, 539, 590, 625, 651, 664, 698
HAP_27	1	108
HAP_28	1	110
HAP_29	10	111, 136, 427, 488, 495, 498, 506, 536, 683, 705
HAP_30	1	117
HAP_31	1	118
HAP_32	5	137, 508, 599, 718, 1165
HAP_33	1	142
HAP_34	4	143, 451, 704, 709
HAP_35	1	145
HAP_36	3	146, 616, 739
HAP_37	6	149, 422, 434, 557, 605, 631
HAP_38	1	423
HAP_39	1	426
HAP_40	1	435
HAP_41	1	436
HAP_42	1	437
HAP_43	1	439
HAP_44	1	440
HAP_45	1	445
HAP_46	1	455
HAP_47	1	459
HAP_48	1	460
HAP_49	1	461
HAP_50	1	462
HAP_51	1	464
HAP_52	2	467, 591
HAP_53	1	473
HAP_54	1	475
HAP_55	1	476

ID	n	voucher
HAP_56	1	479
HAP_57	1	482
HAP_58	1	487
HAP_59	1	490
HAP_60	1	491
HAP_61	1	497
HAP_62	1	500
HAP_63	1	502
HAP_64	1	507
HAP_65	1	511
HAP_66	1	514
HAP_67	2	515, 606
HAP_68	4	520, 580, 642, 686
HAP_69	1	521
HAP_70	1	524
HAP_71	4	525, 610, 673, 737
HAP_72	1	527
HAP_73	1	528
HAP_74	1	531
HAP_75	1	533
HAP_76	1	534
HAP_77	1	535
HAP_78	1	541
HAP_79	1	543
HAP_80	1	544
HAP_81	1	545
HAP_82	1	550
HAP_83	1	551
HAP_84	1	558
HAP_85	1	562
HAP_86	1	565
HAP_87	1	567
HAP_88	1	568
HAP_89	1	570
HAP_90	1	571
HAP_91	1	578
HAP_92	1	579
HAP_93	1	582
HAP_94	1	588
HAP_95	1	593
HAP_96	1	594
HAP_97	1	596
HAP_98	1	601
HAP_99	2	602, 643
HAP_100	1	607
HAP_101	1	608
HAP_102	1	609
HAP_103	1	611
HAP_104	1	612
HAP_105	1	613
HAP_106	1	617
HAP_107	1	618
HAP_108	2	619, 679
HAP_109	1	621
HAP_110	1	624
HAP_111	1	627
HAP_112	1	640
HAP_113	1	657
HAP_114	1	659
HAP_115	1	667
HAP_116	1	674
HAP_117	1	677
HAP_118	1	687
HAP_119	1	688

ID	n	voucher
HAP_120	1	691
HAP_121	1	697
HAP_122	1	700
HAP_123	1	706
HAP_124	1	707
HAP_125	1	714
HAP_126	1	719
HAP_127	1	720
HAP_128	1	764
HAP_129	1	767
HAP_130	1	781
HAP_131	1	782
HAP_132	1	786
HAP_133	1	787
HAP_134	1	788
HAP_135	1	791
HAP_136	1	792
HAP_137	1	794
HAP_138	2	796, 866
HAP_139	1	804
HAP_140	1	805
HAP_141	1	806
HAP_142	1	807
HAP_143	1	808
HAP_144	1	809
HAP_145	1	811
HAP_146	1	812
HAP_147	1	813
HAP_148	1	815
HAP_149	1	816
HAP_150	1	818
HAP_151	1	819
HAP_152	1	820
HAP_153	1	821
HAP_154	1	823
HAP_155	1	824
HAP_156	1	825
HAP_157	1	830
HAP_158	1	842
HAP_159	1	851
HAP_160	1	852
HAP_161	1	855
HAP_162	1	859
HAP_163	1	860
HAP_164	1	861
HAP_165	1	862
HAP_166	1	863
HAP_167	2	864, 868
HAP_168	1	867
HAP_169	1	997
HAP_170	1	999
HAP_171	1	1000
HAP_172	1	1001
HAP_173	1	1002
HAP_174	1	1003
HAP_175	1	1004
HAP_176	1	1005
HAP_177	1	1006
HAP_178	1	1007
HAP_179	2	1008, 1010
HAP_180	1	1009
HAP_181	1	1011
HAP_182	1	1012
HAP_183	1	1130

ID	n	voucher
HAP_184	1	1132
HAP_185	1	1134
HAP_186	1	1135
HAP_187	1	1136
HAP_188	1	1137
HAP_189	1	1139
HAP_190	1	1160
HAP_191	1	1161
HAP_192	1	1162
HAP_193	1	1163
